# Analysis of the largest tandemly repeated DNA families in the human genome

**DOI:** 10.1186/1471-2164-9-533

**Published:** 2008-11-07

**Authors:** Peter E Warburton, Dan Hasson, Flavia Guillem, Chloe Lescale, Xiaoping Jin, Gyorgy Abrusan

**Affiliations:** 1 Dept of Genetics and Genomic Sciences, Mount Sinai School of Medicine, New York, NY 10029, USA

## Abstract

**Background:**

Tandemly Repeated DNA represents a large portion of the human genome, and accounts for a significant amount of copy number variation. Here we present a genome wide analysis of the largest tandem repeats found in the human genome sequence.

**Results:**

Using Tandem Repeats Finder (TRF), tandem repeat arrays greater than 10 kb in total size were identified, and classified into simple sequence e.g. GAATG, classical satellites e.g. alpha satellite DNA, and locus specific VNTR arrays. Analysis of these large sequenced regions revealed that several "simple sequence" arrays actually showed complex domain and/or higher order repeat organization. Using additional methods, we further identified a total of 96 additional arrays with tandem repeat units greater than 2 kb (the detection limit of TRF), 53 of which contained genes or repeated exons. The overall size of an array of tandem 12 kb repeats which spanned a gap on chromosome 8 was found to be 600 kb to 1.7 Mbp in size, representing one of the largest non-centromeric arrays characterized. Several novel megasatellite tandem DNA families were observed that are characterized by repeating patterns of interspersed transposable elements that have expanded presumably by unequal crossing over. One of these families is found on 11 different chromosomes in >25 arrays, and represents one of the largest most widespread megasatellite DNA families.

**Conclusion:**

This study represents the most comprehensive genome wide analysis of large tandem repeats in the human genome, and will serve as an important resource towards understanding the organization and copy number variation of these complex DNA families.

## Background

Tandemly repeated DNA makes up a significant portion of the human genome. Although historically relegated as "junk DNA", tandem repeats have taken on a new importance with the realization that their tandem organization provides potentially unique functional characteristics. Tandemly repeated DNA is organized as multiple copies of a homologous DNA sequence of a certain size (repeat unit) that are arranged in a head to tail pattern to form tandem arrays, and thus represent a distinct type of sequence organization shared by all sequenced genomes. Centromeres from fission yeast to humans contain tandem repeats that are critically important for establishing heterochromatin formation and proper chromosome segregation (reviewed in [1]). Furthermore, tandem repeats have been shown to play a role in paramutation in Maize [2,3] and FWA gene regulation in Arabidopsis [4]. Overall, many of these functions appear to involve RNA interference- mediated chromatin modifications [5,3].

Tandem DNA in the human genome shows a wide range of repeat sizes and organization, ranging from microsatellites of a few base pairs to megasatellites of up to several kb [6,7]. Microsatellites and variable number of tandem repeats (VNTRs) can be highly polymorphic and are important for use as genetic markers. Contraction of a 3.3 kb polymorphic tandem repeat array on chromosome band 4q35 is associated with facioscapulohumeral muscular dystrophy (FSHD) [8-10]. Higher copy number of the salivary amylase (AMY1) gene correlates with protein level in populations with a high starch diet [11]. The Duf1220 protein domain, which is highly expressed in brain, was observed to be amplified specifically in the human lineage and to be undergoing positive selection [12]. And the large macrosatellite array DXZ4 appears to have a unique function in the process of X chromosome inactivation [13]. Thus, tandem repeats play important functional and evolutionary roles in genome biology.

Centromeres of human chromosomes contain the largest tandem DNA family in the human genome called alpha satellite DNA, which has been extensively studied and has emerged as a paradigm for understanding the genomic organization of tandem DNA [14-16]. Its fundamental repeat unit consist of 171 bp monomers, which are found in large highly homologous arrays of up to several million bp at the centromeres of all human chromosomes. These tandem arrays are composed of either diverged monomers with no detectable higher-order structure, or as chromosome-specific higher order repeat units (HORs) characterized by distinct repeating linear arrangements of an integral set of 171 bp monomers [17]. This HOR structure correlates with centromere function [16].

The assembled DNA sequence of most human chromosomes ends abruptly in large gaps in centromeres and heterochromatic regions, often in arrays of diverged monomeric alpha satellite or other tandem repeated "satellite" DNA. To date, only chromosome 8 and the X chromosome end in the higher-order repeat units known to be at these centromeres [17,18]. Furthermore, the large regions of classical heterochromatin such as the those on the long arm of chromosomes 1, 9 and 16, and the short arms of the acrocentric chromosomes 13, 14, 15, 21 and 22 are poorly covered by assembled sequence. These regions are rich in tandemly repeated DNA families, which makes their sequence and assembly difficult. Similarly, the human Y chromosome is rich in repetitive DNA sequences, and required special efforts to obtain a detailed sequence [19].

The following report represents the first overall genome-wide assessment of the number, position and organization of tandem repeats in the sequenced human genome, and therefore represents an important resource for further characterization and overall understanding of genomic organization. An overall genome-wide view of the representation of major tandem repeat families found in the current version of the sequenced human genome (hg18) is important for the study of copy number variation (CNV), because many of these arrays will be highly variable in copy number between individuals. Therefore, bioinformatic analysis was performed in this report using both existing database software and additional searches to extend the databases. The largest most prominent arrays ≥ 10 kb in size currently found in the assembled genome are examined, including large arrays of "simple satellite" sequences such as GAATGn and VNTRs, which reveal unexpected higher-order organization. 96 additional large arrays that are beyond the detection limit of current databases have been identified, which include multicopy gene families and large novel satellite DNA families which display distinct tandem arrangements.

## Results

### Classical satellite repetitive DNA in the human genome

**Figure 1:**
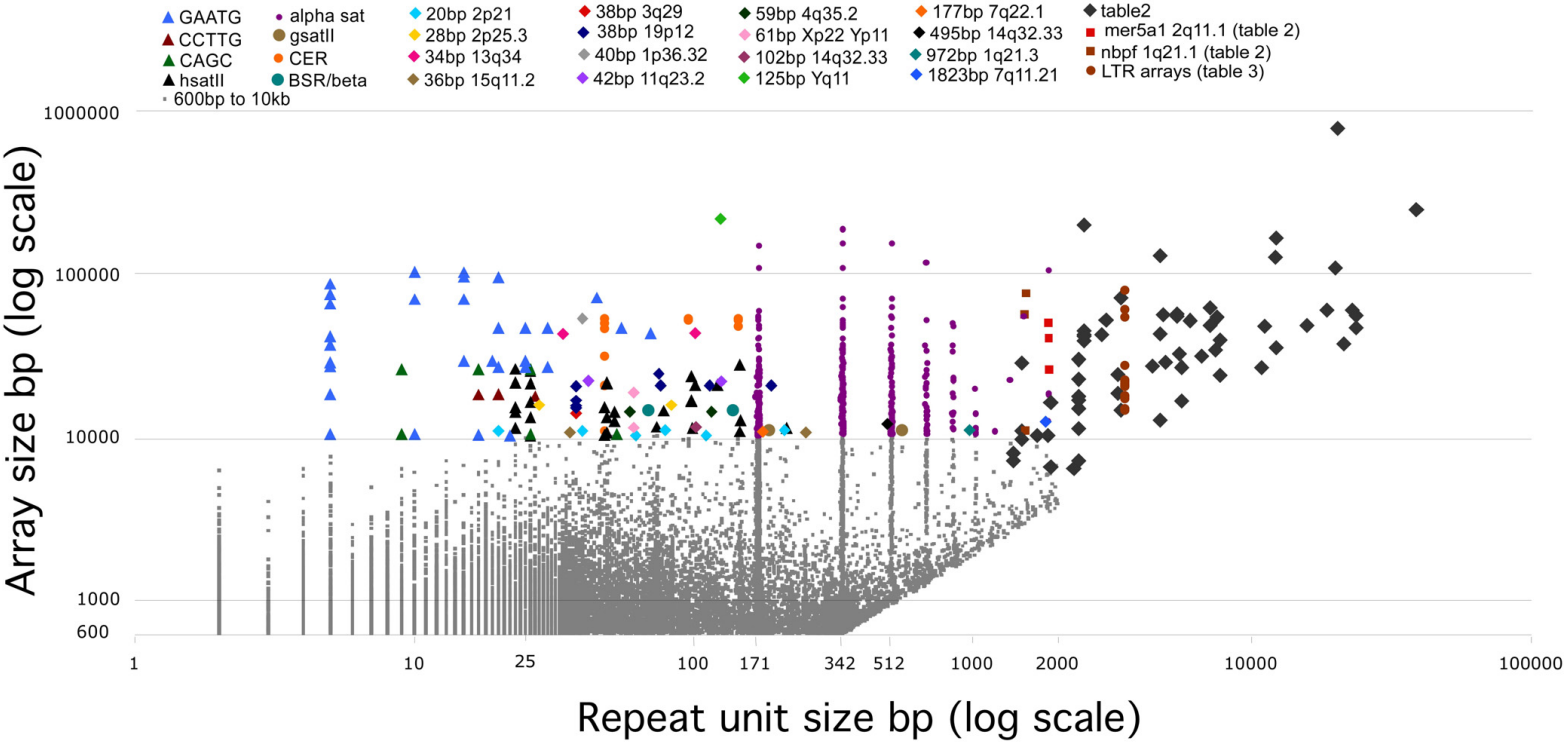
**Analysis of tandem repeats from the human genome**. Output from tandem repeats finder (TRF), plotted showing the repeat unit size on the X axis (log scale) and the array length on the Y axis (log scale). 24,358 arrays between 600 bp and 10,000 bp in length were found (grey squares). 503 arrays ≥ 10 kb found by TRF are shown classified into different types of repeats (see legend at top). Prominent "simple sequence" satellites are shown as color coded triangles. Classical satellites are shown as color coded circles. Single locus VNTR repeats are indicated by color coded diamonds. 373 arrays found at multiples of 171 bp repeat unit size represent alpha satellite DNA (purple circles). Arrays greater then 2 kb not found by TRF are also shown, and listed in Table 2. Some arrays containing repeat units greater than ~1.5 kb are also listed in Table 2 because they contain more complex repeat units than those listed in Table 1. Both the 1.5 kb NBPF repeats (square) and the 1.9 kb "mer5A1" repeats (square) were found by TRF but are listed in Table 2. Multiple LTR arrays (Table 3) are shown as red circles at a repeat unit size of 3.5 kb.

We performed a bioinformatics analysis of the tandemly repeated DNA using the output from tandem repeats finder (TRF) run against hg18 [6], which reports 947,696 arrays containing tandem repeats ranging in size from 2 to 2000 bp [7]. Figure 1 shows these arrays >600 bp plotted by repeat unit size vs. array size (note log scales). In order to examine the largest most prominent tandem repeats in the human genome assembly, the 503 arrays larger than 10 kb were further classified into repeat class (Figure 1). 373 (74%) of these large tandem arrays represented pericentromeric alpha satellite DNA, which showed repeat unit sizes of ~171 bp and multiples (~342 bp, ~512 bp,... 1866 bp), found in arrays as large as 188 kb. Despite this prominence in the TRF dataset, the majority of alpha satellite DNA remains unassembled and is represented by megabased sized centromeric gaps [17,18]. The TRF output (Figure 1) includes some redundancies where the same array is reported more than once as multiples of a basic monomeric repeat unit e.g. 5 bp repeat units can also be reported as arrays of 10, 15, 20 bp repeat units, etc. We therefore compiled all tandem arrays >10 kb found in the human genome sequence (except alpha satellite DNA arrays) (Table 1 and 2), with all redundancies from TRF removed. We cross referenced these arrays with the "simple, low complexity, and satellite" REPEAT MASKER tracks [20] from the UCSC genome browser and their associated entries in the Repbase data base of repetitive elements [21]. Table 1 also includes several large arrays derived by combining multiple smaller (<10 kb) overlapping arrays found by TRF but not highlighted in Figure 1. Table 1 represents the most prominent arrays of satellite sequences currently found in the human genome sequence.

**Table 1:** Arrays of satellite DNA >10 kb in size

**Family**	**Chromo band**	**Repeat Size(s) (bp)**	**Coordinates (hg18)**	**array size (bp)**	**Structure**
GAATG/GAGTG	1q12	5	chr1:141476959–141484530	7,571	
GAATG	4p11	5	chr4:48788006–48853362	65,356	HOR
GAATG	4p11	5	chr4:49328072–49354872	26,800	
GAATG	10p12.33	5	chr10:18881540–18902473	20,933	inversion
GAATG	10p11.1	5	chr10:38812347–38858839	46,492	
GAATG	10p11.1	5	chr10:39116615–39194939	78,302	inversion
GAATG	10q11.1	5	chr10:41674944–41703229	28,286	
GAATG	10p11.21	5	chr10:42110576–42137136	26,560	
GAATG	20q11.21	5	chr20:29267576–29296923	29,347	
GAATG	21p11.2	5	chr21:9795590–9882589	86,855	
GAATG	Yq11.1	5	chrY:12106028–12205425	99,398	HOR, 3360 bp, 5630 bp
GAATG	Yq11.1	5	chrY:12308738–12380225	71,488	
GAATG	Yq12	5	chrY:57228756–57327036	98,280	HOR, 3600 bp
CCTTG	Xp21.2	5	chrX:30716537–30734673	18,136	
CAGC	22q11.1	9, 17, 26	chr22:15419137–15429349	10,212	
CAGC	2p11.2	4, 9, 26	chr2:87486329–87512059	25,730	
hsatII	2p11.2	26, 49	chr2:90958833–90979427	20,594	HO
hsatII	7q11.21	26, 49	chr7:61377290–61396834	19,544	
hsatII	7q11.21	26, 49	chr7:61417257–61440549	23,292	
hsatII	random chr9	26	chr9_random:321296–332315	11,019	
hsatII	random chr9	26	chr9_random:421232–456177	34,945	inversion
hsatII	10p11.1	26, 98	chr10:38915450–38928835	13,385	
hsatII	10p11.1	26, 49	chr10:41703232–41717296	14,064	HOR
hsatII	16p11.2	26, 49	chr16:33783664–33806096	22,432	inversion
hsatII	16p11.2	23, 26	chr16:34038041–34057759	19,718	
hsatII	16q11.2	23, 26	chr16:44943305–45014085	70,780	HOR
hsatII	22q11.1	26	chr22:15227855–15242475	14,620	
Gsat	8q11.1	217	chr8:47404069–47479909	75,840	
Gsat	8q11.1	217	chr8:47356921–47374868	17,947	
Gsat	8q11.1	217	chr8:47119593–47154540	34,947	
GsatX	8p11.1	217	chr8:43535538–43546546	11,008	
GsatII	12p11.1	~200	chr12:34330336–34451537	121,201	Inversions
GsatII	12p11.1	188	chr12:34640002–34652160	12,158	
CER	random chr17	48	chr17_random:135989–174585	38,596	
CER	14q11.1	48	chr14:18267094–18390499	123,405	inversion
CER	18p11.21	48	chr18:15225272–15236086	10,814	
CER	22q11.1	48	chr22:14886892–15006569	119,677	inversion
BSR/beta	Yq12	68	chrY:57392659–57407170	14,511	HOR
hsat4	1q42.13	35	chr1:226783516–226809283	25,767	
hsat4	16p11.1	35	chr16:34775197–34825861	50,664	
Hsat 4/alpha sat	19q12	35	chr19:32423625–32954368	530,743	
Hsat 4/alpha sat	Xp11.1	35	chrX:58228883–58296456	67,573	
VNTR	2p21	20	chr2:43935739–43946526	10,787	
VNTR	2p25.3	28	chr2:1505487–1520958	15,471	
VNTR	13q34	34	chr13:111978814–112021592	47,778	
VNTR	15q11.2	36	chr15:20373884–20384373	10,489	HOR
VNTR	3q29	38	chr3:196680314–196694112	13,798	HOR
VNTR	19p12	38	chr19:20841181–20890996	49,815	
VNTR	1p36.32	40	chr1:2571135–2624075	52,940	HOR
VNTR	11q23.2	42	chr11:113979947–114001592	21,645	
VNTR	4q35.2	59	chr4:188117458–188131626	14,168	
VNTR	Xp22.33 Yp11.32	61	chrX:94937–106217	11,280	HOR
VNTR	Xp22.33 Yp11.32	61	chrX:16203–34821	18,618	HOR, 1600 bp, 2400 bp
VNTR	2q37.1	89	chr2:232395638–232422714	27,076	
VNTR	14q32.33	102	chr14:104767008–104778328	11,320	
VNTR	Yq11.222	125	chrY:20,675,953–20,922,323	246,371	
VNTR	7q22.1	177	chr7:100461970–100472619	10,649	HOR
VNTR	14q32.33	495	chr14:104478982–104490824	11,842	
VNTR	1q21.3	972	chr1:150542280–150553107	10,827	
VNTR	7q11.21	1823	chr7:62846888–62859169	12,281	

**Table 2:** Tandem arrays, repeat units >2 kb

**Chromo Band**	**Array (kb)**	**Unit (kb)**	**Copy**	**% Identity**	**CNV**	**Assembly**	**Gene/TE**	**ref**	**Hg18 coordinates**
***A) Tandem arrays with one gene per repeat unit***									
1q21.1	250.0	38.8	5		+	Gdis, inv	AMY	[11]	chr1:103,893,838–104,143,838
1q21.3	30.4	10.1	3	>90.3%	+		LCE2		chr1:150,897,180–150,927,534
1q23.3	33.9	7.4	4.5	>99%	+		tRNA		chr1:159,675,041–159,708,915
1q42.13	42.1	2.5	16	>99%	-		5sRNA		chr1:226,809,390–226,851,530
4p16.1	42.7	4.7	9	>99%	+	Gdis, inter (1)	DUB3	[32]	chr4:8,935,516–8,978,292
4q35.2	23.8	3.3	7	>99%	--	Inter (2)	DUX4	[10]	chr4:191,224,553–191,248,328
7p14.1	26.9	4.4	5.8	>91%	+		TRGV		chr7:38,347,944–38,374,805
8p23.1	127.0	12.1	10	>98%	+		DEFA		chr8:6,774,101–6,901,753
8q21.2	166.7	12.2	6	>99%	+	Gint	Gor1		chr8:86,744,493–86,911,178
10q26.3	18.4	3.3	6	>99%	--	Inter (2)	DUX4	[10]	chr10:135,328,811–135,347,195
13q21.1	31.1	6.6	5	>99%	+		FLJ40296		.chr13:56,613,947–56,645,084
16p11.1	9.5	1.5	7		+		5sRNA		chr16:34,837,642–34,847,159
17q23.3	59.8	22.9	3	>95%	--		CSH1,2		chr17:59,292,406–59,340,026
Xp22.31	10.1	1.9	5	>95%	+		5sRNA		chrX:9,331,977–9,342,069
Xp11.23	38	2.5	15	>99%	-	Gint	Gage4		chrX:49,059,954–49,271,622
Xq24	56.0	4.8	12	>99%	+		CT47	[31]	chrX:119,893,246–119,948,579
Xq26.3	108.1	19.9	4	>99%	+		CT45		chrX:134,683,931–134,792,078
Yp11.2	791.7	20.3	9	>99%	+	Gint	TSPY		chrY:9,226,249–10,017,916
Yq11.223	55.3	23.6	2.3	>98%	+	Intra (3)	RMBY		chrY:22,069,208–22,124,461
Yq11.223	46.2	23.6	2	>98%	+	Intra (3)	RBMY		chrY:22,431,619–22,477,826
***B) Tandem arrays wholly contained within a gene***									
1q21.1	66.3	1.5	41	>97%	+	Gdis, Gprox, intra (4)	NBPF	[30]	chr1:142,869,841–142,935,173
1q21.1	56.1	1.5	35.7	>95%	+	Intra (4)	NBPF	[30]	chr1:144,022,952–144,079,081
1q21.1	10.8	1.5	6.9	>95%	+	Gprox, Intra(4)	NBPF	[12]	chr1:146,472,125–146,482,922
1q21.3	7.8	1.4	5.6		+		HRNR		chr1:150,452,354–150,460,167
1q32.2	59.4	18.6	3	>97%	+		CR1		chr1:205,769,057–205,828,425
2q11.2	49.5	1.9	26.5	85%	-	SD, intra (5)	FLJ41632	[40]	chr2:95,910,350–95,959,888
2q11.2	39.9	1.9	21.3	85%	+	SD, intra (5)	UNG2430		chr2:97,208,306–97,248,230
2q11.2	25.2	1.9	13.5	82%	+	SD, intra (5)	KIAA 1641		chr2:97,519,639–97,544,901
6q26	26.4	5.6	5	>99%	+	SD	LPA	[32]	chr6:160,953,270–160,979,666
7q22.1	7.0	1.4	5.1	98%	-	SD	BC056606		chr7:99,743,773–99,750,792
8p23.1	47.8	7.1	6.2	>99%	+	SD, intra (6)	AK090418		chr8:7,098,335–7,146,182
8p23.1	61.7	7.1	8	>97%	+	SD, intra (6)	AK090418		chr8:7,606,874–7,668,576
8p23.1	23.5	7.7	3	>97%	+	SD, intra (6)	AK090418		chr8:7,905,630–7,929,101
10p11.21	47.4	11.1	4	>95%	+	SD	ANKRD30A		chr10:37,483,216–37,530,613
10q26.13	42.0	2.9	13	>79%	+		DMBT1	[33]	chr10:124,329,825–124,371,833
19q13.2	48.0	15.7	3	>95%	+		FCGBP		chr19:45,056,041–45,104,055
Yq11.223	17.5	2.4	7		+	Intra (7)	Daz1	[19]	chrY:23,706,609–23,724,156
Yq11.223	29.5	2.4	12		+	Intra (7)	Daz2		chrY:23,783,768–23,813,249
Yq11.223	16.7	2.4	7		+	Intra (7)	Daz2		chrY:23,820,447–23,837,326
Yq11.23	22.3	2.4	9		+	Intra (7)	Daz3		chrY:25,337,948–25,360,227
Yq11.23	22.3	2.4	9		+	Intra (7)	Daz4		chrY:25,409,003–25,431,283
Yq11.23	14.8	2.4	6		+	Intra (7)	Daz4		chrY:25,438,190–25,453,002
Yq11.23	26.2	10.8	2.4		+	Intra (7)	Daz1		chrY:23,726,459–23,752,640
***C) Tandem arrays that contain gene, EST or exon***									
1p36.66	14.4	3.4	4	>98%	+	SD	AK125248		chr1:655,059–669,457
2p13.2	28.4	4.9	5.7	>95%	+		FLJ43987		chr2:73,862,733–73,891,172
4q28.3	41.3	2.5	17	>95%	+	SD, inter (8)	SST	[35]	chr4:132,864,491–132,905,799
5q13.2	36.9	21.3	2	>99%	+	SD	GUSBP		chr5:69,823,338–69,860,219
16p11.2	6.4	1.9	3.4	>88%	+	SD, inter (5)	BC012355		chr16:33,450,421–33,456,850
7p11.2	7.0	2.4	3	>87%	+	SD	Div SST		chr7:57,712,475–57,719,436
17p11.2	11.1	2.4	4	>84%	+	SD	Div SST		chr17:21,825,509–21,836,586
20p11.1	16.1	1.9	8.2	>87%			Div SST		chr20:25,781,895–25,797,997
***D) Megasatellite arrays***									
2q11.2	6.4	1.9	3.4	>89%	-	SD, intra (5)	Mer5A1		chr2:95,986,764–95,993,173
2q37.1	28.1	1.5	18.7		+		Mer20		chr2:232,396,082–232,424,219
4p16.1	16.5	5.6	3	>99%	+		Mer65A		chr4:8,673,058–8,689,560
4p11	51.4	6.0	8	>96%	+	SD, inv, Gprox	Acro		chr4:48,976,229–49,027,623
5p15.1	71.0	3.4	20	>97%	+	SD, Inv	Charlie 2a		chr5:17,570,661–17,641,581
7q36.1	10.1	1.7	5		+	SD	HERVE		chr7:149,361,474–149,371,533
8p23.1	38.8	7.7	5	>98%	+	SD, intra (6)	LTR5A		chr8:7,392,330–7,431,109
8p23.1	12.9	4.7	3	>94%	+	SD, intra (1)	CA	[39]	chr8:7,175,100–7,187,953
8p23.1	12.5	4.7	3	>87%	+	SD, intra (1)	CA		chr8:12,023,084–12,035,607
9q32	32.1	5.5	5.9	>96%	+		L1MA7		chr9:114,860,525–114,892,874
18q22.1	6.3	2.3	3.2	>96%	+		L1PB4		chr18:64,354,361–64,360,677
19p13.2	53.7	7.5	7.5	>97%	+		Charlie 5		chr19:8,708,195–8,761,926
19q13.12	44.5	2.5	18	>95%	-	SD, intra (8)	SST	[35]	chr19:41,448,243–41,492,723
19q13.31	38.5	2.5	16	>95%	-	SD, intra (8)	SST	[35]	chr19:42,451,366–42,489,869
19q13.32	54.4	5.4	10	>98%	+	SD, intra (9)	Mer33		chr19:53,098,557–53,152,942
19q13.33	56.2	5.4	10	>97%	+	SD, intra (9)	Mer33		chr19:55,280,783–55,336,942
Xq23	51.7	3.0	17	>99%	+		DXZ4	[36]	chrX:114,867,433–114,919,088

Assembly- Gdis- Gap at distal end of array, Gprox-Gap at proximal end of array, Gint- Gap internal to array. Inv- inversion within array. SD- array is associated with a surrounding segmental duplication (not including segmental duplications due merely to repeat unit homology within array). Intra- intrachromosomal duplication of related arrays on same chromosome Inter- Interchromosomal duplication of related arrays on different chromosomes. (1) – (9) Related arrays are grouped by number, either intra or interchromosomal, or both, as indicated.

The most abundant tandem repeats after alpha satellite DNA are satellites II and III (Table 1). Satellite III is composed primarily of the pentameric sequence GAATGn (or CATTCn), identified by Repeat Masker as a simple satellite. This family forms a prominent family in the TRF output (Figure 1), with many large arrays at 5 bp and multiples thereof including some repeat units as large as 70 bp, forming arrays of up to ~100 kb. Satellite II is based on highly diverged arrays of GAATG, of which TRF finds 23 bp or 26 bp repeat units and approximate multiples which are identified by Repeat Masker as HsatII, based on comparison to a 59 bp consensus sequence listed in repbase [22]. As expected, prominent arrays of these "classical" satellites are found in the pericentromeric regions of many chromosomes (Table 1). These repeats have been cytologically located to the heterochromatic blocks on chromosomes 1, 9 and 16 [23]. However, in the current assembly these poorly sequenced and assembled regions are riddled with gaps and do not reflect the large amounts of satellite DNA found there. An ~70 kb array of hsatII is seen on the proximal end of the long arm of chromosome 16, directly abuting the heterochromatic gap, and several arrays of hsatII are found in the chromosome 9 random fragments. On chromosome 1, only a small ~7.5 kb array of satellite DNA containing both GAATG and GAGTG is present flanking the hererochromatin of chromosome 1, consistent with satellite III group II which has been previously described there [24]. An additional prominent ~100 kb array of GAATG is found at the distal edge of the Y long arm and is presumably representative of the large heterochromatic block on the Y chromosome.

Additional simple sequence repeats found in arrays larger than 10 kb include 17, 20 and 27 bp repeats identified by TRF consisting of diverged CCTTGn repeats as identified by Repeat Masker, found on Xp11.22. 9, 17, 26 and 53 bp repeats consisting of diverged CAGCn as identified by Repeat Masker were found in arrays on chromosomes 2p11.2 and 22q11.2. To round out the classical satellite DNA is the Centromeric Repeats (CER), based on a 48 bp repeat, which are found in several large arrays on the centromeric q arm of chromosomes 22 and 14, as well as smaller arrays on chromosome 18. Another abundant type of satellite DNA are the three families of gamma satellite DNA (GSAT), a highly diverged relatively GC rich ~200 bp tandem repeat. Large arrays of GSAT listed in repbase as a 704 bp repeat [25], are found at the centromere of chromosome 8 (somewhat interspersed with alpha satellite DNA). GSATX, listed in repbase as a 1205 bp repeat [26], are found at Xp11 and 8q11. GSATII, listed in repbase as a 216 bp repeat, is found in a >100 kb array in 12p11, sometimes mixed with GSATX repeats. Of these Gamma satellites, only the GSATII arrays are found by TRF in arrays >10 kb. An additional satellite repeat is hsat4, based on a 35 bp repeat [16] found in prominent arrays abutting the centromere at chromosome Xp, 16p and 19q, mixed with alpha satellite DNA, and in a 25 kb array just proximal to the 5sRNA repeats in chromosome 1q42.13.

Also apparent from the TRF output are 16 large arrays that consist of locus specific tandem repeats, ranging in repeat size from 20 bp up to 1823 bp. The largest array is the 246.4 kb array (reported by TRF at 218 kb) consisting of DYZ19, a tandem 125 bp repeat first reported by [19] in the euchromatic portion of the Y chromosome (Yq11.222). The inclusion of an array of this size in the finished human genome sequence likely reflects the heroic efforts made in sequencing the Y chromosome. The remaining arrays, which represent classical Variable Number of Tandem repeat (VNTR) arrays, are as large as 50 kb, and do not match known repeat classes as described by Repeat Masker. In several cases, TRF shows redundancy in these VNTR repeats, e.g. the 34 bp VNTR from chromosome band 13q34 is also found as a 101 bp repeat at the same location (Figure 1).

### Higher-order structure in large arrays of satellite DNA

**Figure 2:**
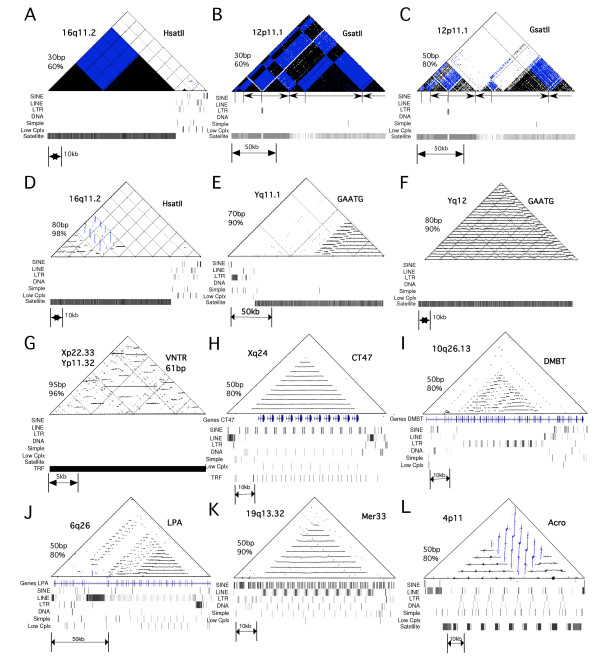
**Dot plot- analysis of tandem arrays reveals higher-order structure**. For each dot-plot shown, the type of repeat, chromosomal location and stringency (window size and % homology) are indicated. Black dots and horizontal lines represent tandem orientation, whereas blue dots and vertical lines represent inverted orientation. The repeat masker tracks for each region are shown below. A-G) Arrays are listed in Table 1. The Repeat Masker tracks indicate a large continuous domain of satellite DNA. A) 70.7 kb array of hsatII from 16q11.2 at low stringency, showing dense pattern indicative of homologous satellite DNA. A large inversion is seen in this array. ~20 kb of neighboring non-satellite DNA is also shown. B) 121 kb array of GsatII from 12p11.1, showing complex multiple inversions within this array. C) Same region as in B at increased stringency, showing 3 distinct domains of homology within overall array. D) Same array as in A at increased stringency, showing higher-order repeats in proximal 50 kb in both orientations. E) ~100 kb array of GAATG on Yq11.1, showing the 3.36 kb higher-order repeats in the distal 60 kb region. F) The 100 kb array of GAATG on Yq12, showing the 3.6 kb HOR across the entire sequenced array. G) The 61 bp VNTR from Xp22.33 at high stringency showing complex higher-order structure. H-L) Arrays listed in Table 2. The Repeat Masker tracks show repetitive patterns containing the different classes of transposable elements. H) The array containing the CT47 genes. I) The DMBT gene, showing the internal repetitive domain structure. J) The LPA gene, showing the internal repetitive domain structure. K) The 54.5 kb array of 5.4 kb megasatellite repeats, each of which contains a Mer33 repeat. L) The 51.4 kb array containing the ~6.0 kb Acro repeats. This array has an inversion in orientation of the repeat units, indicated by the vertical lines visible on the dotplot.

Self-similarity dot plot analysis was performed for all large >10 kb tandem arrays (Table 1) in order to reveal any additional long range repeat unit structure (Figure 2). At low stringency (defined as a 30 bp window with a match of at least 60%), all tandem arrays showed a very dense pattern indicative of the homology between repeat units across the arrays, which was not seen for standard complex DNA at the boundaries of the tandem arrays (e.g. Figure 2A). This analysis revealed the presence of large scale inversions of the orientation of the repeat units in some arrays (Figure 2A, B). As "stringency" is increased (increased window size and % match), this dot plot analysis reveals distinct domains of repeat units with higher similarity within the larger array, shown for the large GSATII array on chromosome 12 (Figure 2C). Remarkably, some "simple sequence" arrays are characterized by highly homologous higher-order repeats (HORs) encompassing part or all of the arrays, as indicated by vertical or horizontal lines that are strongly visible on high stringency dot-plots (Figure 2D–G). The first ~50 kb of the large HsatII array on chromosome 16 shows HORs of ~5.8 kb, with a reversal of orientation (Figure 2D). The distal ~60 kb of the large array of GAATG satellite in the pericentromeric region of the Y chromosome also contains 3360 bp HORs (Figure 2E). And the ~100 kb array of GAATG seen in Yq21 also shows a distinct 3600 bp across the entire sequenced array (Figure 2F). This 3600 bp HOR is consistent with previously reported periodicity of restriction enzyme digests of human DNA [27]. Several of the VNTR arrays also demonstrated HOR structure, such as the 18 kb array of a 61 bp repeat in the pseudoautosomal Xp22.33, which shows irregular HORs of 1.6 and 2.5 kb in a complex arrangement (Figure 2G). These distinct sequence domains and higher-order repeat structures in these "simple sequence" satellite DNA families were only revealed by examination of the large amount of contiguous sequence now available from the human genome.

### Large tandem arrays in the human genome

The TRF analysis is limited to repeats less than 2 kb, and so alternative methods were used to identify tandem repeats larger that 2 kb from the human genome (see materials and methods). This revealed a set of 96 tandem repeat arrays (Figure 1) (Table 2). These represented unique regions which were usually highly visible on the UCSC genome browser as repeating patterns in the Repeat Masker track and in dot plot analyses as characteristic dense patterns of horizontal lines (Figure 2H–L). The vast majority of these were described as regions that showed copy number variation (CNV) according to the databases included in the UCSC genome browser (Table 2), although these regions were not distinguished from other CNVs as containing tandem arrays.

Some repeats that were less than 2 kb were included in Table 2 and shown in Figure 1 because they represented more complex repeats than those listed in Table 1. These included three arrays of the NBPF genes organized in ~1.5 kb repeats (Table 2B), and the largest exon of the hornerin (HRNR) gene characterized by distinct ~1.4 kb repeating units (Table 2B). Arrays of 5sRNA genes from chromosomes 1, 16 and X are included (Table 2A). And several arrays were included because each repeat contains interspersed transposable elements, including a ~1.9 kb repeat characterized by a Mer5A1 element (Table 2D).

10 repeat families were found in multiple arrays, either located on the same chromosome (intrachromosomal) or on different chromosomes (interchromosomal), or both (Table 2). Chromosome band 8p23.1 has the highest concentration of tandem arrays (Table 2), and additional regions with multiple tandem repeats includes chromosome 19q and the Y chromosome, both of which are known to be enriched in tandem repeats [19,28,29].

**Figure 3:**
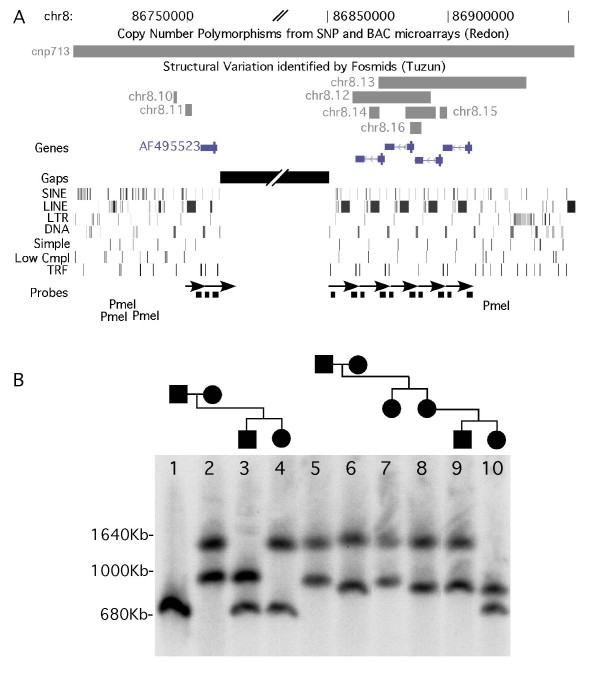
**Analysis of large tandem array in 8q21.2**. A) Information from the UCSC genome browser (hg18) showing region containing the 12 kb tandem repeat from 8q21.2. This repeat array contains an "87 kb" gap with ~5 repeat units on the proximal side and ~1.5 repeats on the distal side. The repeats can be seen in the repeating patterns of the Repeat Masker Tracks. The AF495523 (Gor1) gene is found once in each repeat unit. Copy number variation was detected at this repeat array using both BAC microarrays and fosmids. The restriction enzyme PmeI does not cut in the 12 kb repeats, but cuts close to the edge of the array in the genomic DNA sequence. The position of the PCR amplified probes used on the Southern blot are indicated. B) Pulsed Field Gel analysis of the array size in two pedigrees (lanes 1–4, and lanes 5–10).

Several of these arrays appear in poorly assembled regions, characterized by sequence gaps within the array e.g. the Gor1 repeats in 8q21.2 (Table 2A), or directly abutting them on the proximal or distal side e.g. the NBPF gene family found in several arrays in 1q21.1 (Table 2B) [30]. Furthermore, several arrays may have assembly errors, suggested by the fact that overlap of assembled BAC clones is found precisely at the end of the array or at a change in orientation of the repeat units e.g. the array at 5p15 (Table 2D). These gaps and poor assembly likely indicate significant variation in these regions. In order to further investigate this variation in a repeat array, the Gor1 repeats in 8p21.2 were examined, which are represented in the genome by ~6 copies of a 12 kb repeat that is found on either side of an 87 kb gap (Figure 3A). PFG analysis was performed to assess the size and copy number variation in this repeat, using the PmeI restriction enzyme, which does not cut within the repeat unit and thus releases the whole array in a single large fragment (Figure 3B). A total of 9 arrays in two pedigrees were analyzed. These arrays were polymorphic and spanned a region from 600 kb to 1.7 Mbp, representing ~50–150 repeat units, but were stably transmitted through up to three generations. To our knowledge, this is the largest array of tandemly repeated DNA in the genome after the centromeric alpha satellite arrays.

The large tandem repeat arrays in Table 2 fell into several categories depending on the organization of genes, if any, within them. 20 tandem repeats contain a gene in most or every repeat unit, such that variation in the copy number of the tandem array would change the gene copy number (Table 2A), e.g. the CT47 gene found in 11 precise tandem copies (Figure 2H) [31]. The DUB3 gene is represented in the genome by 9 precise tandem copies (ending at a distal gap), which was previously found in meiotically unstable arrays from 20 to 103 copies [32]. Additional repeat arrays are wholly contained within a gene, such that each repeat unit represents an exon or protein domain (Table 2B), such as the DMBT gene [33] (Figure 2I), the NBPF genes (which contain the duff1220 domain) [12,30], and the LPA gene [34] (Figure 2J). Some additional arrays may contain one or a few genes (or mRNAs or spliced ESTs), and several are contained within an intron of a gene (Table 2C).

**Figure 4:**
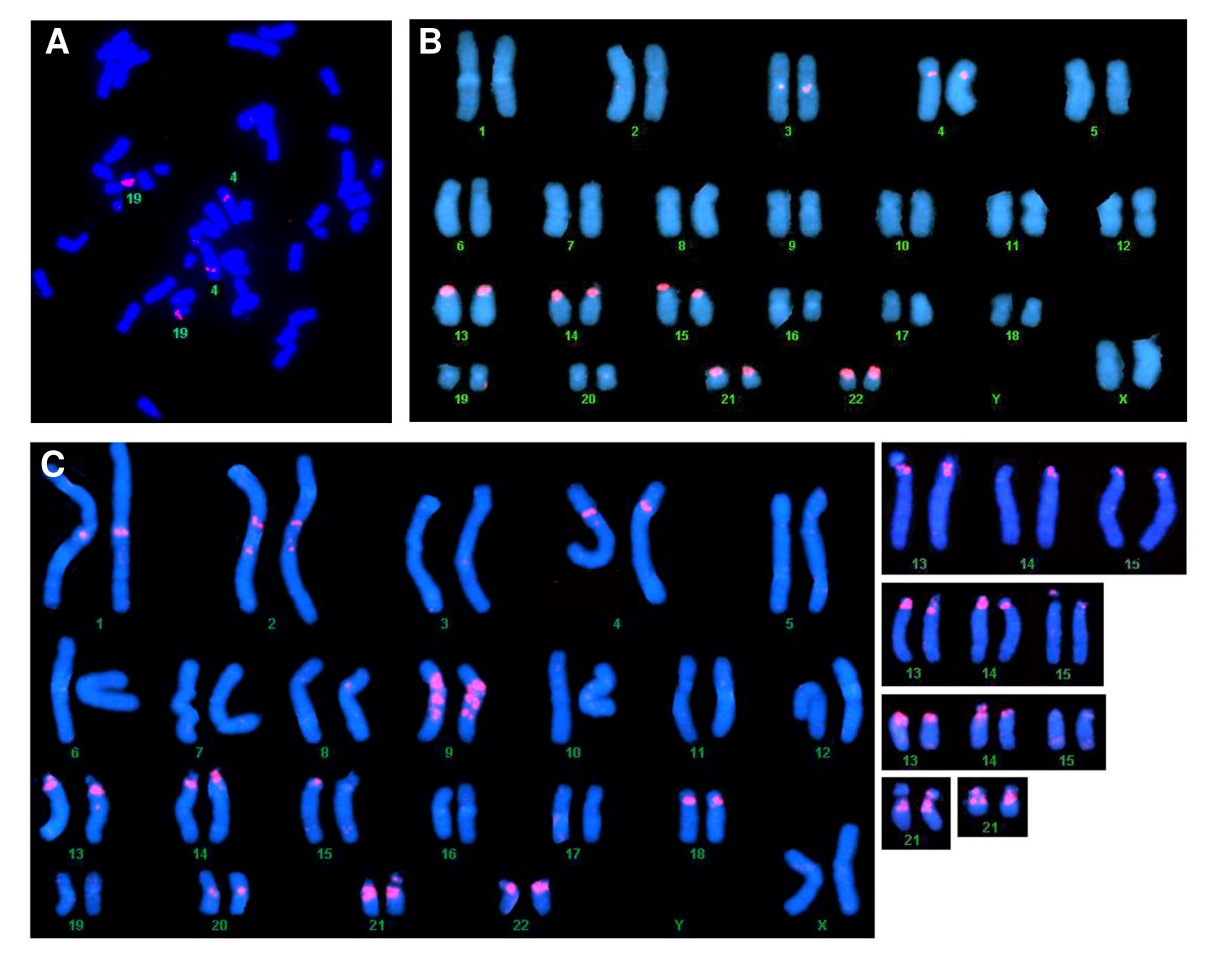
**In situ hybridization of megasatellite DNA families**. A) FISH using a 636 bp probe to the SST repeats from chromosome 4, which hybridizes to chromosome 19 (two arrays, Table 2d) and chromosome 4. B) FISH using a probe from the acro repeats from chromosome 4p11 (Table 2d), which hybridizes to pericentromeric regions of chromosomes 3 and 4, and the acrocentric chromosomes. C) FISH using a probe to the 3.5 kb repeats from the LTR arrays. Right- Additional acrocentric chromosomes from different individuals showing the variation in hybridization patterns.

Other arrays represent large classes of megasatellite DNA which do not contain genes, but are characterized by repeating patterns of interspersed transposable elements (TEs) (Table 2D). Figure 2K shows an example of a 5.4 kb repeat which contains a Mer33 DNA transposon, several LTR retrotransposons, and many SINE and LINE elements, found in two large arrays on chromosome 19q13. Figure 2L shows ~6 kb repeat units from band 4p11 that contains portions of LINEs, LTRs and DNA transposons, as well as ~1.6 kb blocks of the 147 bp Acro satellite DNA identified by REPEAT MASKER. The presence of horizontal and vertical lines on the dot-plot indicates that there is an inversion within this array. FISH probes made from the non-transposon regions between the Acro satellite blocks (Figure 2L) hybridize strongly to the short arms of all acrocentric chromosomes as well as chromosome 4p and 3p (Figure 4B), indicating that it is this entire 6 kb repeat that is amplified on the acrocentric chromosomes. Other large arrays include the SST family of 2.5 kb repeats on chromosomes 4q28.3 and 19q13 (Table 2D)[35], which are listed in Repbase as 1563 bp repeats, with additional diverged arrays on chromosomes 7p11.2, 17p11.2 and 20p11.1. A 636 bp FISH probe made to the region between the 1563 bp "SST" repeats from chromosome 4 hybridizes specifically to the arrays on chromosomes 4 and 19 (Figure 4A). A large polymorphic 3 kb repeat (DXZ4) has been described on the X chromosome in an array of 50–100 copies, which does not contain any Repeat Masker identified TEs [36]. Another striking array at band 2q37.1 consists of 28 kb of diverged 89 bp repeats that are identified by Repeat Masker as a dense block of highly diverged fragments of the DNA transposon Mer20 (consensus 128–219 bp).

### A large tandem DNA family consists of amplified LTR retrotransposons

**Figure 5:**
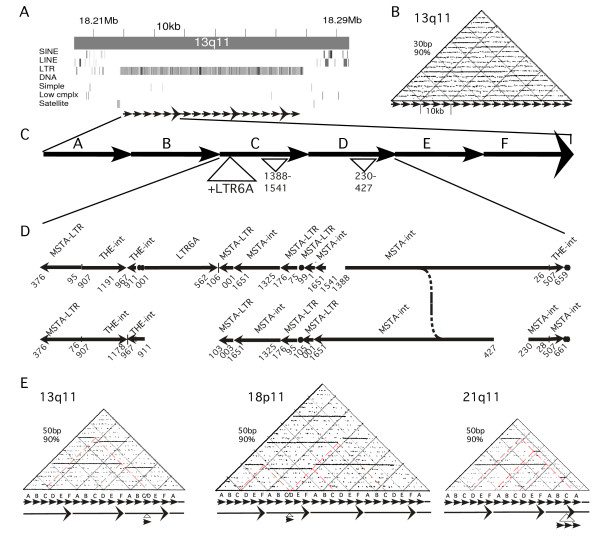
**Analysis of the repeat unit structure of the LTR arrays from chromosomes 13, 18 and 21**. A) Genomic region from chromosome 13q11 containing the LTR array. The REPEAT MASKER Tracks from the UCSC genome browser are shown, which indicate the large 60 kb array of LTR transposons. Homologous monomeric repeat units are indicated by arrows. B) Self similarity dot plot of LTR array. 30 bp windows at 90% homology reveals the ~3.5 kb monomeric repeat units as horizontal lines. C) Schematic of a higher-order repeat unit (HOR) consisting of 6 ~3.5 kb monomeric repeat units. The insertion of an LTR6A into monomer C of each HOR is shown, as well as additional deletions of the MSTA-int repeats. D) Detail of the composition of monomers C and D indicating the MaLR LTR fragments that make up the repeat units, taken from the REPEAT MASKER output and numbered relative to the consensus for each element. The insertion of a full length LTR6A into monomer C can be seen. E) Self-similarity dot plots of LTR array from chromosome 13q11, 18p11 and 21q11 at 50 bp windows 90% homology. The HOR organization is revealed as bold solid horizontal lines, and are shown schematically by arrows below. Putative unequal crossing over events unique to each LTR array are revealed by the gaps and shift of these lines, and deleted monomeric repeat units indicated below.

One unique megasatellite family was observed which consists entirely of the MaLR class of LTR retrotransposons. This family is organized into ~3.5 kb monomeric repeat units containing fragments of both MSTA and THE-1 LTRs and internal open reading frames (MSTA-ints) in both orientations (Figure 5, Table 3), which have undergone expansion into a tandem array. These "LTR arrays" are found on 9 different chromosomes in 25 arrays, covering ~460 kb of DNA (Table 3), and account for a significant portion of the MSTA LTR transposons in the genome. These arrays appear as solid blocks of LTR retrotransposons in the UCSC genome browser (Figure 5A), with 10 distinct arrays on chromosome 9, and the three largest arrays in the pericentromeric regions of chromosomes 13q, 18p and 21q (Table 3). FISH analysis using probes designed to conserved regions of these LTR array repeats reveals the 25 arrays, including small arrays seen in the genome browser with less than 2 repeat units (not included in Table 3) on chromosomes 9q22.1, 3q29, and 2q21 as well as additional arrays on chromosomes 14, 15, 20, and 22 (Figure 4C). Thus, this family is the largest most widespread megasatellite family yet described, and represents a novel arrangement and expansion of the abundant MaLR class of LTR retrotransposons.

**Table 3:** LTR arrays in the human genome

**Chromo band**	**Array size (kb)**	**Repeat units**	**Chromo position**
1q12	14.7	4	chr1:141,614,596–141,629,292
1q12	14.7	4	chr1:142,011,066–142,025,761
1q12	14.4	4	chr1:142,245,576–142,259,928
2q11	21.7	8	chr2:94,794,007–94,815,544
4p11	17.8	5	chr4:48,911,896–48,929,687
4p11	14.8	4	chr4:49,259,299–49,274,079
8p11.1	17.0	5	chr8:43,489,114–43,506,146
9p13.3	20.3	7	chr9:33,567,377–33,587,718
9p13.1	20.5	7	chr9:38,536,498–38,557,002
9p11.2	27.2	9	chr9:42,429,334–42,456,540
9p11.2	27.2	9	chr9:43,025,353–43,052,560
9p11.2	22.1	7	chr4:43,964,543–43,986,620
9q12	22.1	7	chr9:66,111,804–66,133,881
9q12	21.0	7	chr9:68,742,783–68,763,792
9q22.33	17.5	5	chr9:98,936,138–98,953,601
13q11	60.4	18	chr13:18,218,666–18,279,040
18p11.21	79.5	25	chr18:14,244,667–14,324,176
21q11.2	53.8	16	chr21:14,145,756–14,199,554

FISH analysis of 8 unrelated individuals showed no variation in the presence of LTR arrays on non-acrocentric chromosomes, but revealed significant variation in the presence and number of LTR arrays on the acrocentric chromosomes (Figure 4C). A centromeric array was seen on all 16 chromosome 13s examined, 7 of which also had an array on the short arm. A centromeric array was also seen on all 16 chromosome 21s examined, 4 of which also has a short arm array, 1 of which had a centromeric and a slightly more distal second array, and 3 of which had all three arrays. Seven chromosomes 14 had only a short arm array, 3 had only a centromeric array, while 5 had both and 1 had no detectable array. Six of the chromosome 15s examined had only a short arm array, and 10 had no detectable array. Thus, the acrocentric chromosomes show up to three arrays resolvable by FISH (short arm, centromeric, and more distal) and a large amount of variability in the population. The presence of LTR arrays at the centromeres of chromosomes 13 and 21 in the genomic sequence are consistent with these results.

These LTR arrays are in general embedded in large interrelated segmental duplications, which on chromosomes 13, 18 and 21 are complex highly related mosaics several hundred kb in size (data not shown). The large LTR arrays from chromosomes 13, 18 and 21 are each characterized by higher order repeat (HOR) structures consisting of 6 ~3.5 kb monomeric repeats (Figure 5C), with distinct patterns of MSTA fragments repeated once per HOR (Figure 5D). In particular, each HOR on chromosomes 13, 18 and 21 is marked by insertion of another type of LTR transposon, a full length LTR6a (Figure 5C, D). These HORs are readily visible as bold solid horizontal lines on dotplots (Figure 5E). The dispersal of these HORs likely occurred due to the interchromosomal segmental duplications between chromosomes 13,18 and 21. However, the HORs from each chromosomal array shows unique rearrangements (Figure 5E). On chromosome 13, the third HOR is missing a 3.5 kb monomeric repeat unit, presumably due to unequal crossing over between HORs (as shown in figure 5D). The array on chromosome 18 has a similar crossover event, but the monomeric repeat was deleted from the second HOR (Figure 5E). And the array from chromosome 21 has a 3 monomer deletion in the third HOR. Thus, these unique HOR rearrangements on each array suggest that unequal crossing over events have been ongoing since the dispersal of the arrays to each chromosome.

## Discussion and conclusion

Tandemly repeated DNA represents a unique class of DNA in the human genome with unusual sequence organization in regular repeat units. We have presented here genomic analysis of the largest tandem arrays in the human genome (Figure 1). For example, analysis of the largest arrays of simple sequence "satellite" DNA in the genome (Table 1) revealed unexpected higher-order structure, including inversions, domains of homology, and extensive HOR structures (Figure 2A–G). HORs in "simple" sequence have been suggested by restriction enzyme periodicities seen as large bands on Southern blots e.g. [27], but the size and extent of these HORs and the diverse sizes seen here for several different classes of satellites has not been previously described. Elucidation of such structures was only possible because of the large (up to several hundred kb) contiguous sequences available from the human genome sequence. The HOR structure of human centromeric alpha satellite has been implicated as important in centromere function [16]. By analogy, it is possible that the simple satellites that display HOR and domain structures (Table 1) may represent a subclass involved in chromosome function.

We have also examined arrays that contain larger repeats, generally greater than 2 kb, with some monomeric repeat units as large as 40 kb (Table 2). Tandem DNA represents an important source of copy number variation because the number of repeat units can vary by several fold at individual arrays, which for these large arrays can represent Mbps of DNA sequence. We have shown, for example, that the size of a tandem array consisting of 12 kb repeats at chromosome 8q21.2 can vary from 600 kb to 1.7Mbp, even though these were represented in the genome as 5–6 repeats spanning an "87 kb" gap. Many of the repeats listed in Table 2 have been observed to be found in much larger polymorphic arrays than represented in the genome sequence, such as the Dux4 genes which are associated with FSHD [8,9]. Moreover, the majority of the tandem repeats are found in regions with detectable copy number variation, usually by performing aCGH with BAC arrays or sequence analysis of fosmids [37,38] and found in databases for human CNVs. However, these detection methods such as aCGH do not clearly distinguish these large tandem arrays, which may vary by hundreds of copies, from other types of CNVs that are found in fewer copies, such as insertions and deletions. Ideally, analysis of the array size and copy number at each individual allele would be desirable, in order to distinguish between, for example, two medium size arrays versus one large and one small array, which may have significant phenotypic differences. Currently, PFG gel analysis as shown in Figure 3 has been used to distinguish individual alleles of large tandem DNA arrays.

Chromosome band 8p23 contains the highest concentration of large tandem repeats, which occur in the two repetitive proximal and distal regions (RepP and RepD, respectively) which flank a large 4.7 Mbp polymorphic inversion [39,40]. A 7 kb repeat found in 4 different arrays of up to 8 repeat units were seen in opposite orientations in RepD, and homologous single copies of this repeat are seen in RepP (data not shown). Two additional arrays consisting of 4.7 kb repeats are seen in both RepP and RepD, and share homology with the Dub3 array on 4p16 [32,41]. Whether these tandem repeats contribute to, or are a consequence of, the common inversions and duplications seen in 8p23 is not known.

The large tandem repeats listed in Table 2 have been categorized according to whether they contain genes or are contained within genes. 20 arrays contain repeated genes with one gene per repeat unit (Table 2A). In these cases, variation in copy number of the tandem repeat would lead to different numbers of genes, which may effect dosage of the protein product and lead to phenotypic changes, such as observed for the AMY1 gene [11]. 23 repeat arrays were wholly contained within genes (Table 2B), such that each repeat unit contained one or more exons representing protein domains [12]. In these cases, a change in copy number may represent a change in the number of these repeated domains present in a protein product, which may have a strong effect on function and phenotype. 8 other arrays were more difficult to categorize (Table 2C). Some appeared to contain single genes (or spliced ESTs or mRNAs), including small ncRNA sequences. Others showed the repeat array contained entirely within an intron, which may represent a source of microRNAs involved in gene regulation e.g. see FLJ43987 (Table 2C).

17 additional tandem arrays that did not contain genes were categorized as megasatellites because they are made up of repeat units that contain interspersed TEs. These regions presumably represent regions of the genome that accumulated TEs in a normal fashion over time, and were subsequently amplified into large tandem repeats. Each is distinguished by striking repetitive patterns in the REPEAT MASKER tracks from the UCSC genome browser and distinct dot plots containing dense patterns of horizontal and/or vertical lines (Figure 2H–L). Several arrays appear in the REPEAT MASKER tracks as large dense blocks of LTR or DNA transposons. For example, the large 246 kb array of 125 bp repeats in Yq11.222 [19] appears in the LTR track as a solid block of 49 copies of highly diverged fragments of LTR12B. The 667 bp consensus of LTR12B listed in Repbase has within it 3 tandem copies of an ~125 bp repeat which is ~85% similar to the 125 bp repeat units seen in this array, and thus it seems possible that this 125 bp repeat is derived from a hugely expanded portion of a LTR12B sequence. Another large array on chromosome 2q37 consists of 89 bp repeats with ~80% homology to positions 128–219 of the DNA transposon mer20. The beginning of this array contains a full length mer20 (positions 1–219) in the same orientation, and thus this array likely represents tandem expansion of the latter 89 bp of this LTR transposon into the large array.

The LTR arrays (Figure 4C, Figure 5) also illustrate novel composition, organization, and evolutionary processes in a tandem repeat. These arrays consist entirely of the MaLR class of LTR transposons, including both LTRs and internal open reading frames of the MSTA elements, organized into 3.5 kb monomeric repeat units. These have been dispersed to over 25 arrays on ~12 chromosomes, including the acrocentric chromosomes with significant variation for the presence of arrays in both the short arm and pericentromeric regions (Figure 4C). On chromosomes 13, 18 and 21, the available DNA sequence reveals distinct HOR structure consisting of 6 monomeric repeat units, each of which contain an insertion of an LTR6A element (Figure 5C). The dispersal to multiple chromosomes may be related to the large segmental duplications in which these arrays are embedded. However, unequal crossing over events have occurred within these HORs since this dispersal to multiple chromosomes. Thus, we can putatively order a series of events that shaped these arrays, 1) the transposition of the MaLR elements and amplification into the 3.5 kb monomeric repeat units, 2) insertion of the LTR6a element into one of the monomers, 3) expansion into HORs containing 6 monomers 4) dispersal to chromosomes 13, 18 and 21 and 5) unequal crossing over in the HORs on each chromosome.

In summary, we have compiled the collection of largest tandem arrays from the sequenced human genome. We have demonstrated unexpected secondary higher-order structure in "simple sequence" DNA. We have added to current databases the list of tandem arrays with repeat units greater than 2 kb, including novel megasatellites that contain amplified patterns of interspersed transposable elements. The tandem repeats described in this paper represent an important resource for understanding the genomic organization of the human genome.

## Methods

### Analysis of tandem repeats in the human genome

Tandem Repeat Finder was run on the human genome hg18 at high sensitivity, and the primary results found on tandem repeats database (TRDB) [6]. Large repeats were examined in the UCSC genome browser, and repeats were identified according to their assignment in the simple, low complexity, or satellite tracks of REPEAT MASKER [20], according to their match in RepBase [21]. For larger repeats, a script was performed that identified >250 regions where at least three highly similar interspersed repeats from Repeat Masker SINE, LINE, LTR or DNA tracks were found at a similar distance from each other, set at a limit of ≥ 1.5 kb. These were further examined using the UCSC genome browser or by dot plots, and those that contain tandem repeats were identified and included for further study (Figure 1, Table 2). Self-similarity dot plots were performed using MacVector pustell DNA matrix.

### Pulsed Field Gel Electrophoresis

PFG gels, Southern blotting, and hybridization were performed using standard protocols [42]. Briefly, cultured human EBV transformed lymphoblastoid cells were embedded in agarose plugs and digested with proteinase K in 1% Lauryl sarcosine 0.5 M EDTA pH 9.5. Restriction enzyme digestion was performed by equilibration of agarose plugs in restriction enzyme buffer at 4 degrees and addition of restriction enzyme (PmeI). PFG gels (Figure 3) were run on a Biorad CHEF DRII using ramped pulsed times from 120–320 seconds at 150 V for 46 hours, in a 1% agarose gel in 0.5× TBE buffer, and Southern blotted. Probes were designed from within the 12 kb repeat units using PCR primers from conserved regions, which amplified a 2 kb fragment (Gor1AL- TGGATGATCTGTGCCAGGTA and Gor1AR- AAGGAGAAGTCCCACCCAGT) and a 1.6 kb fragment (Gor1BL- GGGTAGAGGAGGGTGAAAGG and Gor1BR- TTGGAACCATGCGAGTGATA, which were designed in "unique" sequence and avoided any Repeat Masked sequences.

### In situ hybridization

PCR primers were designed to conserved regions of repeat units using primer 3 . PCR products to be used as FISH probes were designed to include only "unique" regions which did not include any interspersed repetitive elements as identified by REPEAT MASKER. PCR amplification of the tandem DNA arrays using conserved PCR primers will amplify multiple repeat units. Purified PCR products were labeled by nick translation incorporating biotin-dUTP. Fluorescent In Situ Hybridization (FISH) was performed using standard protocols [43]. For FISH analysis of LTR arrays, at least 5 metaphases were analyzed per person from primary lymphocyte cultures and were karyotyped using DAPI banding. PCR primers used to amplify FISH probes for LTR arrays were designed to two conserved regions of the 3.5 kb repeat units, and complementary PCR primers were designed that amplified the entire repeat unit in two ~1.7 kb fragments. PCR primers were Pair 1: 1A:CTGTACCTGTGCATCTTTC and 2B:GGGAGTAGCCTGCTGCAGAGG and the complementary pair 2A:GAAAGATGCACAGGTACAG and 1B:CCTCTGCAGCAGGCTACTCCC. To PCR amplify FISH probes for analysis of the repeat on 4p11 (Figure 4B), primers pairs that encompass 1.1 kb from right side of the acro repeat to left side of mer21 repeat (4p11-bL GCTGGGTGATGGCAGTAAGA 4p11-bR ATCTGGAGCCACACCTTGAT) were used.

## Authors' contributions

PEW, DH, FG, CL, XJ AND GA performed experiments and analyzed data, and PEW wrote the paper.
